# Mitochondrial fission regulator 2 (MTFR2) promotes growth, migration, invasion and tumour progression in breast cancer cells

**DOI:** 10.18632/aging.102442

**Published:** 2019-11-18

**Authors:** Guanming Lu, Yuanhui, Lai, Tiantian Wang, Weihao Lin, Jinlan Lu, Yanfei Ma, Yongcheng Chen, Haiqing Ma, Ruilei Liu, Jie Li

**Affiliations:** 1 Department of Breast and Thyroid Surgery, Affiliated Hospital of Youjiang Medical University for Nationalities, Basie, Guangxi, China; 2 Department of Breast and Thyroid Surgery, Eastern Hospital of the First Affiliated Hospital of Sun Yat-sen University, Guangzhou, Guangdong, China; 3 Department of Breast and Thyroid Surgery, Shandong Provincial Hospital Affiliated to Shandong University, Ji’nan, Shandong, China; 4 Department of Breast and Thyroid Surgery, First Affiliated Hospital of Sun Yat-sen University, Guangzhou, Guangdong, China; 5 Department of Stomatology, Affiliated Hospital of Youjiang Medical University for Nationalities, Basie, Guangxi, China; 6 Department of Oncology, Fifth Affiliated Hospital of Sun Yat-sen University, Zhuhai, Guangdong, China; 7 Department of Breast and Thyroid Surgery, Third Affiliated Hospital of Sun Yat-sen University, Guangzhou, Guangdong, China; 8 Division of Thyroid and Parathyroid Endocrine Surgery, Massachusetts Eye and Ear Infirmary, Harvard Medical School, Boston, MA 02114, USA

**Keywords:** breast cancer, MTFR2, survival, glycolysis

## Abstract

Introduction: Mitochondrial fission regulator 2 (MTFR2) belongs to the MTFR family, and 2 isoforms of MTFR2 are produced by alternative splicing. The role of MTFR2 in breast cancer (BC) remains unknown.

Results: MTFR2 was upregulated in BC tissues and was strongly associated with tumor characteristics. Moreover, Kaplan-Meier and Cox proportional hazards analyses indicated that high MTFR2 expression was related to poor overall survival. In addition, the capacity for migration and invasion decreased in two BC cell lines after knockdown of MTFR2. The epithelial-mesenchymal transition pathway was inhibited in MTFR2-silenced cells. MTFR2 can switch glucose metabolism from OXPHS to glycolysis in a HIF1α- and HIF2α-dependent manner.

Conclusion: Taken together, our results indicate that increased expression of MTFR2 is associated with tumour progression in breast cancer cells through switching glucose metabolism from OXPHS to glycolysis in a HIF1α- and HIF2α-dependent manner.

Materials and methods: We obtained data from The Cancer Genome Atlas (TCGA) and the Gene Expression Omnibus (GEO) to analyse MTFR2 expression in BC. The prognostic value of MTFR2 expression was assessed using the Kaplan-Meier method. The biological influence of MTFR2 on BC cell lines was studied using proliferation, Transwell migration, invasion and mitochondrial function assays.

## INTRODUCTION

Breast cancer (BC) is one of the most common cancers worldwide and the second leading cause of cancer-related mortality in women [1]. Although there are various treatment options, including surgery, endocrine therapy and chemotherapy, the prognosis of patients diagnosed with this type of cancer is still poor [2–4]. Recent studies have identified many oncogenes and signalling pathways that are associated with the progression and occurrence of BC [5, 6]. However, the mechanisms underlying the progression of BC remain unknown. Consequently, it is very important to explore additional molecular biomarkers that can serve as novel prognostic biomarkers and lead to new treatments for BC patients.

The shape of the mitochondrial network results from the cumulative activity of two opposing processes: fusion and fission [7]. These processes collaborate to ensure the homeostatic maintenance of mitochondrial function, the bioenergetics of the cell, and the commitment to mitosis [8]. Mitochondria are multifaceted organelles that are at the centre stage of energetics/metabolism, but mitochondria can also take part in cellular redox balance, calcium balance, lipid modification, and cell death [9]. Recent studies have revealed that abnormal mitochondrial fission is involved in the pathogenesis of many diseases and that it contributes to the progression of tumours [10, 11]. The function of the mitochondria depends on continuous fission-fusion events and can influence several cell functions, such as calcium buffering and apoptosis [12]. Some studies have demonstrated that unstable mitochondrial fission is a driver of the progression of malignancies [13, 14]. In addition, a significantly high rate of mitochondrial fission occurs in chemoresistant cells, and mitochondrial fission could determine chemotherapy sensitivity [15, 16]. Mitochondrial fission regulator 2 (MTFR2), also called family with sequence similarity 54, member A (FAM54A), is poorly studied in tumours [17, 18]. Some studies have reported that MTFR2 plays an important functional role in mitochondrial and aerobic respiration and that it promotes mitochondrial fission in cells [17, 19]. A recent study revealed that MTFR2 was significantly elevated in glioma samples and that higher MTFR2 expression could be correlated with poor prognosis. The loss of MTFR2 could decrease cell growth and the tumourigenesis of cells. However, the expression and biological functions of MTFR2 in BC remain unclear.

In this study, we aimed to analyse the clinicopathological features of MTFR2 expression in BC patients and evaluate its biological functions in BC tumours. Our results demonstrate for the first time that MTFR2 is upregulated in BC tumours and that MTFR2 expression could predict poor survival. The silencing of MTFR2 expression could repress BC cell proliferation, migration and invasion. Taken together, these findings suggest that MTFR2 could serve as a new therapeutic target in breast cancer.

## RESULTS

### MTFR2 is upregulated in human BC tissues and serves as an independent prognostic marker in BC patients

**Figure 1:**
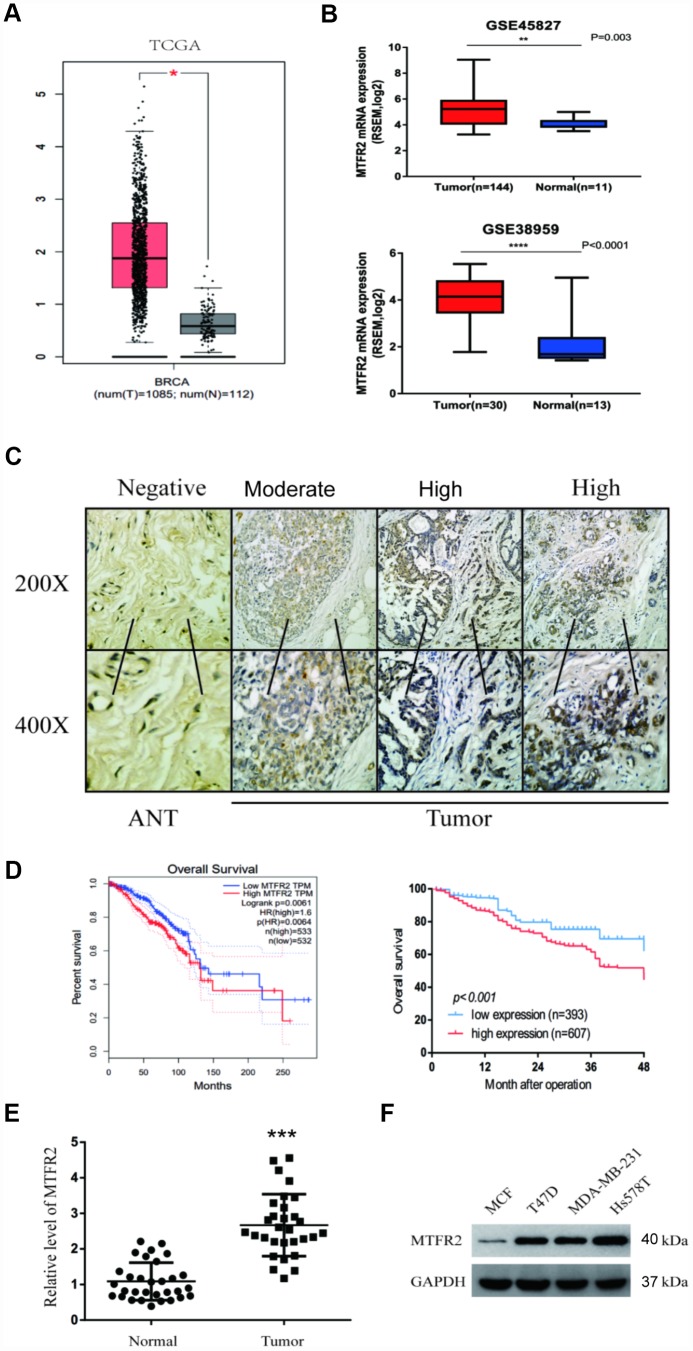
**MTFR2 was upregulated in BC and negatively correlated with prognosis.** (**A**) The relative level of MTFR2 in the TCGA database (Student’s two-tailed paired test * p<0.05). (**B**) The relative level of MTFR2 in the GSE45827 and GSE 38959 databases (Student’s two tailed paired test * p<0.05, ** p<0.01, *** p<0.001). (**C**) IHC staining of MTFR2 in BC samples. (**D**) The overall survival analysis of the TCGA database and our own database (Log rank test). (**E**) The relative level of MTFR2 in our own database (Student’s two tailed paired test *** p<0.001). (**F**) Western blot of MTFR2 in the BC cell line.

To examine MTFR2 expression in BC, we used mRNA sequencing or microarray datasets from TCGA and GEO. We compared 1085 BC tissues and 112 normal tissues from TCGA, and the results showed that MTFR2 expression was significantly higher in tumour tissues than in normal tissues (*p*<0.01) (Figure 1A). In addition, we analysed MTFR2 expression from the GSE38959 (*p<*0.001) and GSE45827 (*p*=0.003) datasets, and the results indicated that MTFR2 expression was significantly elevated in BC tissues compared with normal tissues (Figure 1B).

We then performed IHC to investigate MTFR2 protein expression levels in 1000 pairs of BC tissues and the corresponding ANTs. The MTFR2 immunostaining signals revealed that 607 BC patients exhibited high levels of MTFR2 protein expression and that MTFR2 protein expression was significantly higher in BC tissues than in ANTs (Figure 1C, *p*=0.016). We then analysed the correlations between MTFR2 expression and the clinicopathological features of BC patients. The results are shown in Table 1. Our findings revealed that the expression of MTFR2 was strongly associated with age (*p=*0.001), tumour grade (*p*=0.009), lymph node metastasis (*p*=0.010) and HER2 status (*p=*0.016) (Table 1).

**Table 1:** Correlation between MTFR2 expression and clinicopathological characteristics of BC.

**Clinicopathological variables**	**n**	**MTFR2 expression**		***P* Value**
		**Low (393)**	**High (607)**	
Age, years				
<50	490	166	324	**0.001**
≥50	510	227	283	
Tumour Size, mm				
<20	523	198	325	0.354
≥20	477	195	282	
Tumour grade				
1, 2	647	235	412	**0.009**
3	353	158	195	
Venous involvement				
Negative	914	359	555	0.963
Positive	86	34	52	
Lymph node metastasis				
Negative	432	150	282	**0.010**
Positive	568	243	325	
ER			
Negative	253	96	157	0.610
Positive	747	297	450	
PR			
Negative	411	166	245	0.556
Positive	589	227	362	
HER2			
Negative	618	261	357	
Positive	382	132	250	**0.016**

Abbreviations: ER, oestrogen receptor; PR, progesterone receptor; HER2, human epidermal growth factor receptor 2.

Kaplan-Meier analysis was used to examine the prognostic value of MTFR2 expression. The results indicated that BC patients with higher MTFR2 expression levels had lower overall survival (OS) rates than those with low MTFR2 levels (*p*<0.001) (Figure 1D). We then analysed the OS rates from the GEPIA dataset. The results revealed that patients with high MTFR2 expression levels exhibited lower OS than those with low MTFR2 expression levels (*p*=0.0064) (Figure 1D). Multivariate analysis demonstrated that MTFR2 expression (HR, 1.96; 95% CI, 1.55-2.48; *p*=0.03) and lymph node metastasis (HR, 1.91; 95% CI, 1.43-2.56; *p*=0.02) were independent prognostic factors for OS (Table 2). We then measured MTFR2 mRNA levels in 30 pairs of tissues and corresponding ANTs using qRT-PCR and found that the level of mRNA expression of MTFR2 was higher in BC tissues than in ANTs (Figure 1E). Taken together, these results indicated that MTFR2 mRNA is more highly expressed in BC tissues than in ANTs.

**Table 2:** Univariate and multivariate Cox regression analysis of risk factors associated with overall survival.

**Variables**	**Univariate analysis**			**Multivariate analysis**		
	**HR**	**95% CI**	***P* Value**	**HR**	**95% CI**	***P* Value**
MTFR2 expression (High vs. Low)	1.82	1.45−2.27	**<0.01**	1.96	1.55−2.48	**0.03**
Age (≥50 vs. <50)	2.24	1.87−3.33	0.07			
Tumour Size (<20 vs. ≥20)	2.78	1.24−3.03	0.26			
Venous involvement (negative vs. positive)	1.11	0.77−3.83	**<0.01**	1.62	1.25−2.09	0.39
Lymph node metastasis (negative vs. positive)	3.18	3.60−9.87	**<0.01**	1.91	1.43−2.56	**0.02**
ER (negative vs. positive)	0.76	0.21−1.17	0.09			
PR (negative vs. positive)	1.63	0.69−4.01	0.08			
HER2 (negative vs. positive)	2.76	1.39−4.86	**<0.05**	1.80	0.65−3.04	0.05

Abbreviations: ER, oestrogen receptor; PR, progesterone receptor; HER2, human epidermal growth factor receptor-2.

### MTFR2 promotes the proliferation, migration and invasion of breast cancer cells

**Figure 2:**
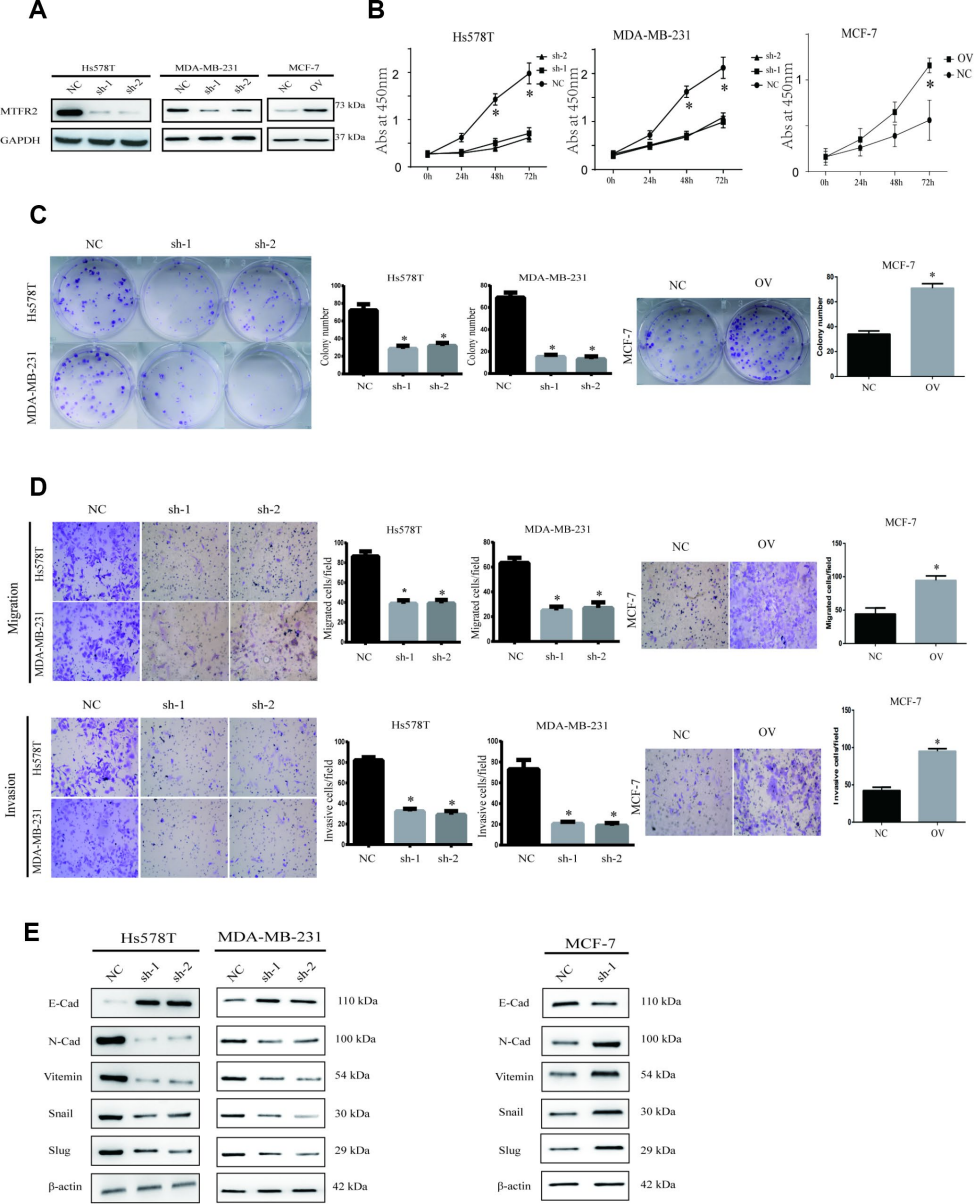
**MTFR promotes the proliferation, migration and invasion of BC.** (**A**) Western blot of MTFR2 in the cell line (NC, Negative Control; OV, overexpression; Sh, small hairpin RNA). (**B**) The CCK-8 assay of different cell lines (Student’s two one-tailed paired test * p<0.05). (**C**) The colony formation assay and statistical analysis of different cell lines (Student’s two one-tailed paired test * p<0.05). (**D**) The migration and invasion assays of different cell lines (Student’s two one-tailed paired test * p<0.05). (**E**) Western blot of EMT markers of different cell lines.

To uncover the bio-function of MTFR2 in BC cells, we analysed MTFR2 expression in BC cell lines. Except for MCF-7, the cell lines expressed high levels of MTFR2 (Figure 1F). MTFR2 was stably knocked down in the Hs578T and MDA-231 cell lines and overexpressed in the MCF-7 cell line (Figure 2A). Colony formation assays and CCK-8 assays revealed that higher levels of MTFR2 showed higher proliferation rates in breast cancer cell lines (Figure 2B, 2C). We next detected the effect of MTFR2 on cell migration and invasion motility. The results revealed that the capability for cell migration and invasion significantly increased in cells with relatively high levels of MTFR2 (Figure 2D). These results suggest that MTFR2 promotes proliferation, migration and invasion in BC cells.

### MTFR2 promotes the epithelial-mesenchymal transition of BC cells

Our study has revealed that MTFR2 promotes proliferation, migration and invasion in BC cells. The EMT phenotype is associated with invasion in cancer cells [20]. The results showed that mesenchymal markers such as N-cadherin, Snail, Vimentin and slug decreased, but epithelial markers such as E-cadherin increased in the MTFR2 knockdown cell lines; however, mesenchymal markers increased, but epithelial markers decreased at both the RNA and protein levels in the MTFR2-overexpressing cell line (Figure 2E). These results suggest that MTFR2 promotes the mesenchymal transition of BC.

### MTFR2 maintains the aerobic glycolysis of BC cells

**Figure 3:**
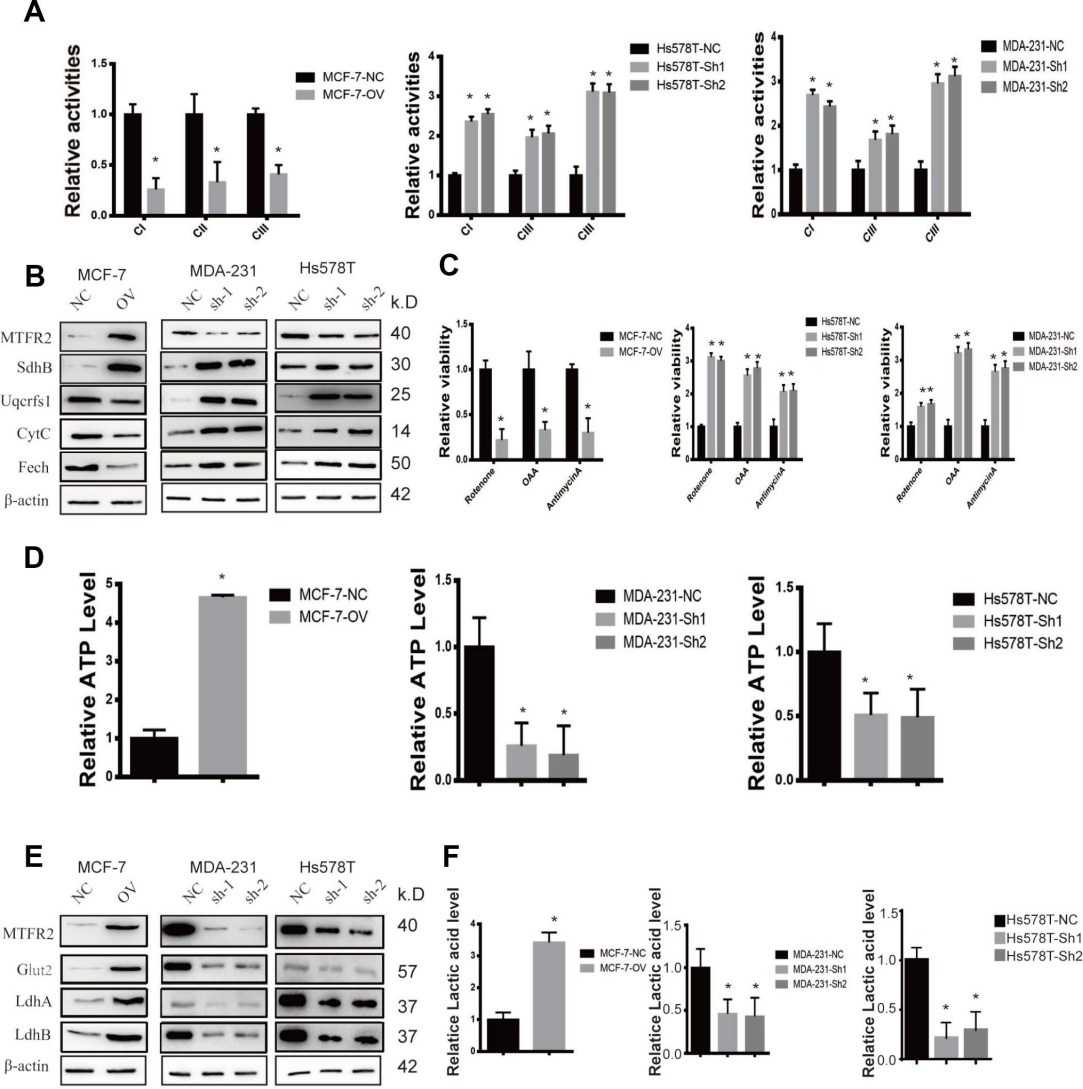
**MTFR promotes the glycolysis of BC.** (**A**) The relative activities of the CI CII and CIII of different cell lines (Student’s two one-tailed paired test * p<0.05). (**B**) Western blot of OXPHOS markers of different cell lines. (**C**) The relative viability of different cell lines treated with different inhibitors (Student’s two one-tailed paired test * p<0.05). (**D**) The relative ATP level of different cell lines (Student’s two one-tailed paired test * p<0.05). (**E**) Western blot of glycolysis markers of different cell lines. (**F**) The relative lactic acid level of different cell lines (Student’s two one-tailed paired test * p<0.05).

MTFR2 has rarely been studied in tumourigenesis. However, previous evidence showed that MTFR2 was correlated with mitochondrial function. In our study, we found that the activities of mitochondrial complexes I, II and III significantly increased in sh-MTFR2 cells (Figure 3A p<0.001), which is consistent with the levels of the Fe-S-containing subunits Ndufs1 (complex I), SdhB (complex II), and Uqcrfs1 (complex III) (Figure 3B p<0.001). Furthermore, other mitochondrial proteins, such as CytC (cytochrome C) and Fech (ferrochelatase), were also increased in MTFR2 knockdown cells. In contrast, we found that the mitochondrial complexes and proteins of Ndufs1, SdhB, Uqcrfs1, CytC and Fech decreased in MTFR2-overexpressing cells (Figure 3B p<0.001).

To further detect the relationship of MTFR2 and mitochondrial function, we detected the viability of MTFR2 knockdown and overexpression cells under various inhibitors of mitochondrial complexes. The results showed that the viability of MTFR2 knockdown cells significantly increased while the viability of MTFR2-overexpressing cells decreased compared with NC (Figure 3C p<0.001). Then, we found that the level of ATP significantly decreased in MTFR2 knockdown cells and increased in MTFR2-overexpressing cells (Figure 3D p<0.001). The process of ATP production is involved in OXPHOS and glycolysis. Therefore, we detected the levels of several proteins involved in glycolysis, such as Glut1 (glucose transporter 1) and LdhA/B (lactate dehydrogenase A/B). The results showed that these proteins were significantly decreased in MTFR2-knockdown cells and increased in MTFR2-overexpressing cells (Figure 3E p<0.001). Furthermore, we measured the content of lactic acid and found that MTFR2 knockdown cells produced and secreted less lactic acid. In contrast, the levels of lactic acid were increased in MTFR2-overexpressing cells compared with NC cells (Figure 3F p<0.001).

**Figure 4:**
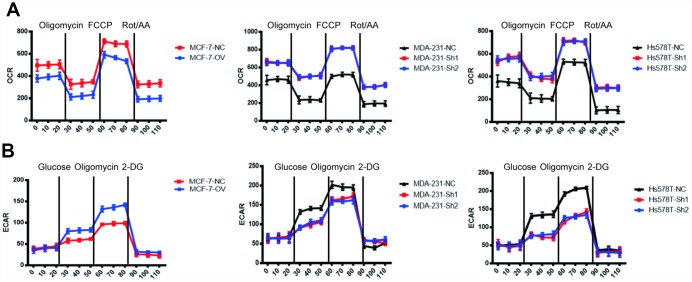
**MTFR switches the OXPHOS of BC to glycolysis.** (**A**) The OCR of different cell lines. (**B**) The ECAR of different cell lines.

To verify this hypothesis, we detected the OCR (oxygen consumption rate) and the ECAR (extracellular acidification rate) to measure mitochondrial respiration and glycolytic rate by using an Agilent Seahorse analyser. The results demonstrated that MTFR2 knockdown cells had higher resting OCR and OXPHOS after treatment with carbonyl cyanide p-(trifluoromethoxy) phenylhydrazone (FCCP) than before treatment (Figure 4A p<0.001). Moreover, MTFR2 knockdown cells had a much lower ECAR after treatment with glucose and oligomycin than before treatment. Taken together, these results suggest that MTFR2 maintains cellular aerobic glycolysis. (Figure 4B p<0.001)

### MTFR2-induced Hif1α and Hif2α promote proliferation, migration and invasion in breast cancer cells

**Figure 5:**
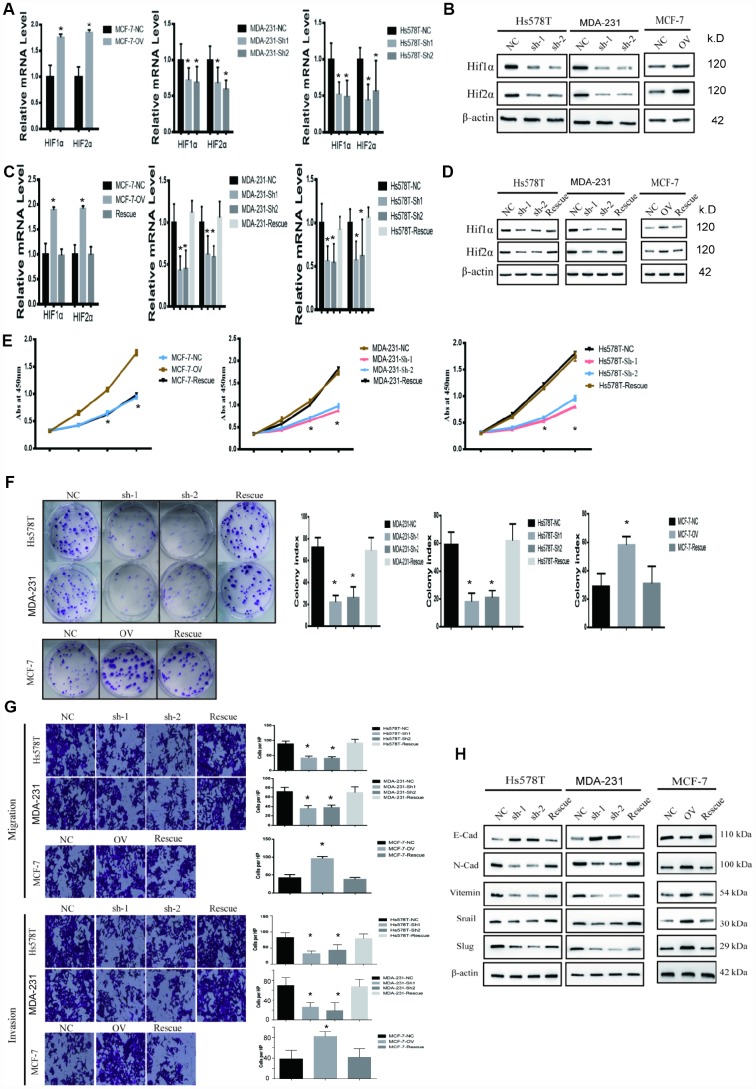
**MTFR promotes the proliferation and migration and invasion of BC in a HIF1α- and HIF2α-dependent manner.** (**A**) The relative mRNA levels of HIF1α and HIF2α in different cell lines (Student’s two one-tailed paired test * p<0.05). (**B**) Western blot of HIF1α and HIF2α in different cell lines. (**C**) The relative mRNA levels of HIF1α and HIF2α in different cell line (Student’s two one-tailed paired test * p<0.05). (**D**) Western blot of HIF1α and HIF2α in different cell lines. (**E**) CCK-8 assay of different cell lines (Student’s two one-tailed paired test * p<0.05). (**F**) The colony formation assay and statistical analysis of different cell lines (Student’s two one-tailed paired test * p<0.05). (**G**) The migration assay and invasion assay of different cell lines (Student’s two one-tailed paired test * p<0.05). (**H**) Western blot of EMT markers of different cell lines.

We further investigated the mechanism by which MTFR2 affects OXPHOS and glycolysis. HIF is a key factor in regulating the levels of genes involved in glycolytic respiration. In our study, we found that MTFR2 knockdown resulted in a significant reduction in Hif1α and Hif2α. However, MTFR2 overexpression resulted in significant production of Hif1α and Hif2α (Figure 5A, 5B p<0.001). To further confirm the critical role of HIF1α and Hif2α in mitochondrial functions, we constructed a rescue cell line; we overexpressed both HIF1α and Hif2α in the MTFR2 knockdown cell line and knocked down both HIF1α and Hif2α in the MTFR2-overexpressing cell line (Figure 5C, 5D p<0.001). We next applied proliferation, Transwell, and invasion chamber assays. MTFR2 promoted the proliferation, migration and invasion of BC cells, which corresponded to the previous study, and the function of the rescue cells was totally restored compared with the NC cells (Figure 5E–5G p<0.001); the EMT markers were also totally restored (Figure 5H p<0.001), indicating that MTFR2 exerts its function in a HIF1α- and Hif2α-dependent manner.

### MTFR2-induced Hif1α and Hif2α are responsible for maintaining aerobic glycolysis

**Figure 6:**
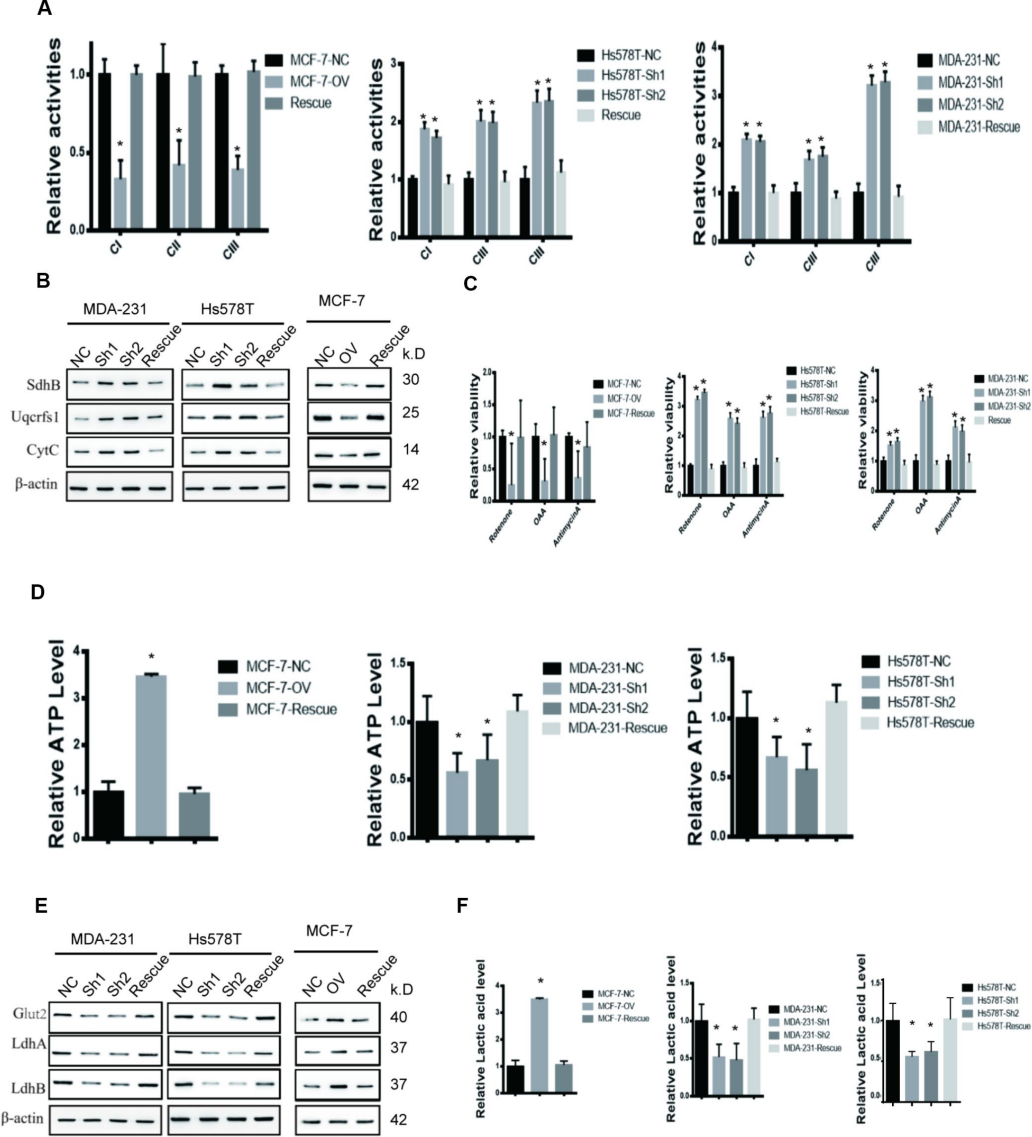
**MTFR promotes the glycolysis of BC in a HIF1α- and HIF2α-dependent manner.** (**A**) The relative activities of the CI CII and CIII of different cell lines (Student’s two one-tailed paired test * p<0.05). (**B**) Western blot of OXPHOS markers of different cell lines. (**C**) The relative viability of different cell lines treated with different inhibitors (Student’s two one-tailed paired test * p<0.05). (**D**) The relative ATP level of different cell lines (Student’s two one-tailed paired test * p<0.05). (**E**) Western blot of glycolysis markers of different cell lines. (**F**) The relative lactic acid level of different cell line (Student’s two one-tailed paired test * p<0.05).

**Figure 7:**
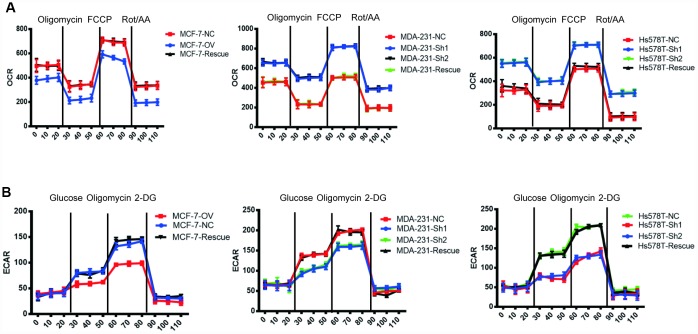
**MTFR switches the OXPHOS to the glycolysis of BC in a HIF1α- and HIF2α-dependent manner.** (**A**) The OCR of different cell lines. (**B**) The ECAR of different cell lines.

We have previously shown that MTFR2 promotes proliferation, migration and invasion through switching from OXPHOS to aerobic glycolysis. We further studied the OXPHOS function and aerobic glycolysis function in the cell lines described above. The OXPHOS activity markers were totally restored compared with NC in both MDA-231 and Hs578T cell lines (Figure 6A and Figure 6B p<0.001). The reaction of cells to the inhibitors was also totally restored (Figure 6C p<0.001). ATP production decreased in the MCF-7 rescue cell line but increased in the MDA-231 and Hs578T cell lines (Figure 6D p<0.001). The glycolysis markers Glut2, LdhA and LdhB were restored to baseline and lactic acid production, which is an important glycolysis marker restored (Figure 6E and Figure 6F p<0.001). We next applied OCAR and ECAR assays. The results show that the rescue cell line totally returned to baseline compared with the NC cell line (Figure 7A, 7B p<0.001). Taken together, the results show that MTFR2 switches glucose metabolism from OXPHS to aerobic glycolysis in a HIF1 α - and Hif2α-dependent manner, the correlation between MTFR2 and HIF1α and HIF2α in TCGA database was detected in Supplementary Figure 1, however, the correlation didn't quite match, it may due to the complicated network in metabolism, other gene may exert its' function through HIF1α and HIF2α, not only MTFR2.

### MTFR2 promotes the proliferation and metastasis of breast cancer cells *in vivo*

**Figure 8:**
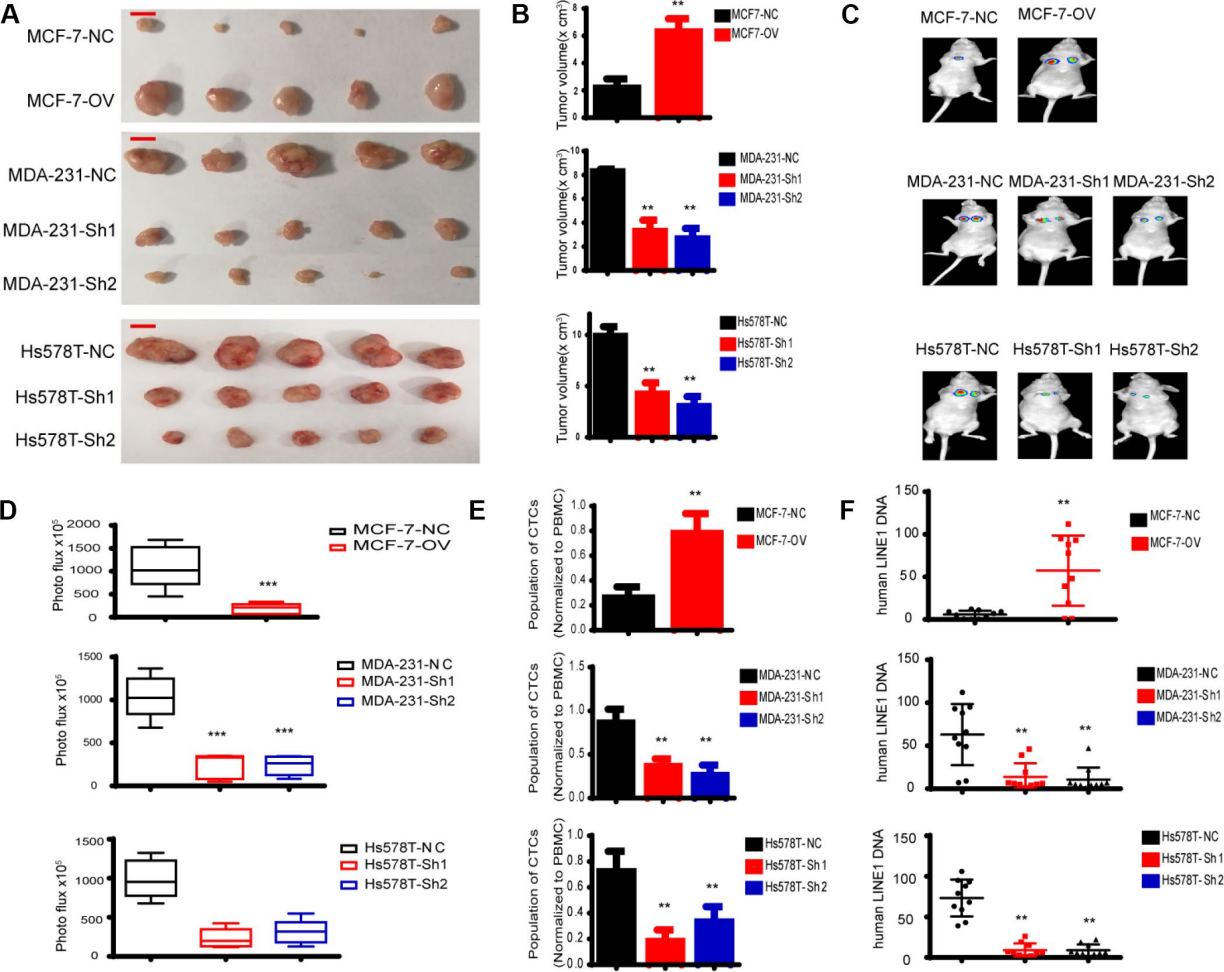
**MTFR promotes the progression of breast cancer cells *in vivo.*** (**A**) A representative image of the tumours of xenografted mice, scale bar 0.5 cm. (**B**) The tumour volume of xenografted mice (Student’s two one-tailed paired test * * p<0.01). (**C**) A representative image of lung metastasis. (**D**) Photo flux of lung metastasis (Student’s two one-tailed paired test * ** p<0.001). (**E**) The population of CTCs (Student’s two one-tailed paired test * * p<0.01). (**F**) The human LINE1 DNA level (Student’s two one-tailed paired test * * p<0.01).

We have indicated that MTFR promotes the proliferation and migration and invasion of breast cancer *in vitro.* To further uncover the biological function, we established a xenograft mouse tumour model and a lung metastasis model. As shown in Figure 8A, 8B, the tumour volume of MCF-7-OV significantly increased compared with the control, whereas knockdown of MTFR in MDA-231 and Hs578T cells inhibited tumour growth. Lung metastasis significantly increased in cells with higher levels of MTFR (Figure 8C, 8D p<0.001). Metastasis is a complicated process that includes early invasion and late colocation into target organs. We analysed the circulating tumour cells (CTCs) from whole blood samples. The results were normalized to peripheral blood mononuclear cells (PBMCs). The results show that the population of CTCs increased in MCF-7-OV, MDA-231-NC and Hs578T-NC cells (Figure 8E p<0.01). We also analysed the human LINE1 DNA level, another indicator of CTCs, normalized to mouse LINE1 DNA levels. Cells with relatively high levels of MTFR harbour relatively high levels of human LINE1 DNA (Figure 8F p<0.01).

## DISCUSSION

To our knowledge, this study is the first to explore MTFR2 expression in BC. Our results provide evidence that the expression level of MTFR2 is higher in BC tissues than in ANTs. In addition, IHC revealed that the expression of MTFR2 was significantly correlated with the clinicopathological features of BC patients and with patient prognosis. Furthermore, our data showed that the overexpression of MTFR2 could promote breast cancer cell progression. Moreover, the results showed that the inhibition of MTFR2 suppressed the ability of BC cells to proliferate, migrate and invade. The findings of these analyses suggest that MTFR2 promotes BC cell progression and might be a novel predictor for BC patient prognosis.

MTFR2 plays an important functional role in mitochondria and promotes mitochondrial fission [19]. Many studies have explored whether mitochondrial dysfunction is implicated in tumourigenesis and whether mitochondrial fission is associated with breast carcinoma invasion and metastasis [21, 22]. We hypothesized that MTFR2 was upregulated in BC tissues and could serve as a biomarker for BC. First, we analysed MTFR2 expression in TCGA datasets and microarray data from GEO. In addition, we measured MTFR2 expression in 30 pairs of BC specimens and ANTs using qRT-PCR. Taken together, the results suggest that MTFR2 expression levels are higher in BC tissue than in the corresponding ANTs. Furthermore, we performed IHC to detect MTFR2 expression in 1000 pairs of BC samples and ANTs. Further evaluation of the relationship between MTFR2 staining and patient clinical characteristics showed that the MTFR2 protein level in BC tissues is associated with age, tumour grade, lymph node metastasis and HER2 status. A previous study demonstrated that tumour grade, lymph node metastasis, HER2 status and other clinicopathological parameters are important in BC progression and metastasis and affect the prognosis of BC patients [23, 24] Furthermore, a Kaplan-Meier survival analysis indicated that the OS of patients with high levels of MTFR2 expression was significantly lower than that of patients with low levels of MTFR2 expression, and a Cox proportional analysis strongly indicated that MTFR2 might be useful as an independent prognostic marker for OS.

Mitochondria are critical for cancer metabolism due to their synthesis of ATP by oxidative phosphorylation [25]. Many studies have revealed that mitochondrial homeostasis is critical for cellular functions including growth, division, energy metabolism and cancer migration [26, 27]. The shape of the mitochondrial network results from the cumulative activity of two opposing processes: fusion and fission [7]. Some studies have revealed that mitochondrial fission proteins could promote the cell cycle, cell proliferation, cell invasion and cell migration [28]. In addition, mitochondrial fission is associated with apoptosis [29]. Moreover, some studies have revealed that increasing mitochondrial fission could promote mitophagy [30, 31].

Cancer cells degrade glucose to produce lactate even in an oxygen-enriched environment, which is known as glycolysis, also known as the Warburg effect. This phenomenon tends to be involved in proliferative cells, including cancer cells, and it is also called the cell-autonomous reprogramming of cancers [32]. Many studies have shown that cancer cells can adapt to the restricted microenvironment through metabolic reprogramming, such as hepato-carcinoma [34]. HCC cells can re-modulate the metabolic strategy to produce more ATPs to sustain proliferation and produce more Malonyl-CoA for use in the environment lacking amino acids [33, 34]. A handful of the enzymes contribute to the reprogramming of metabolism, including the well-known enzymes IDH1, IDH2 and fumarate hydratase (FH). These enzymes were identified as transforming enzymes due to their persistent activation. Another well-studied enzyme is PKM2, an isoform of the pyruvate kinase, which is regulated by oncogenic signalling. The latest study argues against PKM2 as a good therapeutic target for most cancers. Controversies arose when understanding why glycolysis was predominant in cancer cells. The controversies can be divided into two parts. First, more ATP. Studies have shown that cancer cells tend to degrade glucose into lactic acid to produce more ATP in a shorter time. However, ATP is produced more abundantly through the TCA cycle than through glycolysis. Second, the one-carbon unit is one of the most important units that glycolysis can produce and is critical for the synthesis of nuclear acids and amino acids. Lactic acid is secreted to the cell suspension through glycolysis, and many carbon units are lost.

Epithelial-mesenchymal transition is a revised cellular programme by which epithelial cells transform into mesenchymal cells. EMT is important in embryogenesis such as tissue morphogenesis. EMT is reported in tumourigenesis in multiple types of cancers. It is reported to be involved in the initiation state of tumourigenesis, and cancer cells with stem-like properties are more likely to undergo EMT than other cancer cells [35]. When EMT is activated, epithelial cells lose cell polarity and cell junctions. The expression of certain cytoskeletal proteins, such as E-cadherin, is lost, and transcription factors, such as the ZEB family and SNAI family, are activated. Cancer cells degrade the extra cellular matrix and more easily migrate and invade. Several signalling pathways have been reported to be involved in EMT, such as TGFβ. The activation of the SMAD family triggers EMT and subsequent migration and invasion cascades. The canonical Wnt pathway is considered a key activator of EMT. Activation of the Wnt pathway allows more β-catenin to enter the nucleus and exert its function as a transcription factor. The ZEB family and SNAI family are the targets of Wnt and are critical for the activation of EMT. NOTCH exerts its function as β-catenin. When activated, it enters the nucleus and promotes the transcription of EMT transcription factors [36].

Taken together, our research showed that MTFR promotes growth, migration, invasion and tumour progression in breast cancer cells by promoting EMT through the modulation of metabolism from OXPHOS to glycolysis. It may be an ideal therapy target in the future.

## MATERIALS AND METHODS

### High-throughput data processing

Data on the expression of MTFR2 in BC were obtained from The Cancer Genome Atlas (TCGA, https://gdc.cancer.gov/), which is a public database. The data used in the microarray were based on the Affymetrix platform and were downloaded from the Gene Expression Omnibus (GEO) datasets GSE38959 and GSE45827 (https://www.ncbi.nlm.nih.gov/geo). The data from TCGA were log_2_ transformed, and the results were analysed using Excel and GraphPad Prism 7.0 software.

### Patient information and clinical sample collection

In total, 1000 paired BC tissue samples and adjacent normal tissues (ANTs) were obtained from BC patients who underwent mastectomies at the First Affiliated Hospital of Sun Yat-sen University (Guangzhou, China), the Third Affiliated Hospital of Sun Yat-sen University (Guangzhou, China), the Eastern Hospital of the First Affiliated Hospital of Sun Yat-sen University (Guangdong, China) and the Shandong Provincial Hospital affiliated to Shandong University (Shandong, China) between January 2013 and December 2014. All BC tissue samples were confirmed by pathology, and the cancer molecular subtype was determined according to oestrogen receptor (ER), progesterone receptor (PgR), and HER2 status, according to the guidelines of the 15^th^ St. Gallen International Breast Cancer Conference, 2017 [37]. The specimens were stored in RNAlater solution (Invitrogen, USA) immediately after resectioning. Then, all specimens were frozen in liquid nitrogen and stored at −80°C. Overall survival (OS) was defined as the period between surgery and death or last contact. This study was approved by the Ethics Committee of the First Affiliated Hospital of Sun Yat-sen University, the Third Affiliated Hospital of Sun Yat-sen University and Youjiang Medical University for Nationalities and conformed to the 1964 Declaration of Helsinki and its later amendments or comparable ethical standards. Clinical samples were collected from patients after written informed consent was obtained.

### Quantitative real-time polymerase chain reaction (qRT-PCR)

Total RNA was extracted with Trizol reagent (Invitrogen, NY, USA). qRT-PCR was performed using the SYBR Green PT-PCR detection system (Takara, Japan). β-actin was used as the control. The relative mRNA expression levels were quantified using the 2^−ΔΔCt^ method. All quantitative real-time PCR experiments were performed in triplicate. The primer sequences used in qRT-PCR were MTFR2 forward, 5′-GAAACTGGATCCCAATGTGAA-3′ and reverse 5′-GAATAAGGTTAAGCTTCGTGCAA-3′ and GAPDH forward 5′-TGTGGGCATCAATGGATTTGG-3′ and reverse 5′-ACACCATGTATTCCGGGTCAAT-3′.

### Immunohistochemistry (IHC) and antibody detection

Tissue specimens were fixed in formalin and embedded in paraffin for MTFR2 immunohistochemistry (IHC). The MTFR2 antibody for IHC was obtained from Abcam (ab236978). After deparaffinization, hydration and blocking, a primary anti-MTFR2 rabbit polyclonal antibody was added and incubated with the specimens overnight at 4°C. Finally, all sections were assessed by comparing the degree of staining between each paired BC and normal specimen under a microscope. MTFR2 expression was evaluated by the IHC-based score, which is composed of the positive cell score and staining intensity score. Staining intensity was rated on a scale of 0-3, where 0=negative, 1=weak, 2=moderate, and 3=strong. The total IHC score was condensed into the following four levels: 0 for a total score=0, 1 for a total score=1-100, 2 for a total score=101-200, and 3 a for total score=201-300.

### Cell culture and cell transfection assay

Human mammary cancer cell lines T47D, MCF-7, Hs578T and MDA-MB-231 were obtained from the American Type Culture Collection (ATCC, Manassas, VA). Hs578T, MCF-7 and T47D were cultured in Dulbecco’s Modified Eagle Medium (DMEM), Minimum Essential Medium (MEM) and RPMI-1640, respectively, and supplied with 10% foetal bovine serum (Life Technologies, Grand Island, NY, USA), penicillin G, streptomycin and amphotericin B. Lentivirus shRNAs were constructed by Shanghai Generay Biotech Co., Ltd. MTFR2 was cloned into the expression vector pCMV (Invitrogen) for overexpression. The transfection assay was performed using Lipofectamine 2000 (Invitrogen, CA, USA) according to the manufacturer’s protocol at approximately 50–70% cell confluence.

### Western blotting

Cells were washed twice with PBS and then lysed in cold RIPA buffer with protease inhibitors. Twenty micrograms of total protein was transferred to a nitrocellulose membrane after being denatured in a 10% SDS-PAGE gel for 90 min. After transferring for 1 h, the membranes were blocked with 5% nonfat milk in Tris-buffered saline containing 0.1% Tween-20 (TBST) for 1 hour at room temperature. The membranes were then incubated overnight with 4% primary antibodies, washed three times with TBST and then incubated with secondary antibodies (anti-rabbit IgG) for 1 h at room temperature. The membranes were washed three times with TBST, and then the target proteins were detected using the ECL (EMD Millipore, MA, USA) method. Western blotting was performed using antibodies directed against MTFR2 (1:500, Abcam), E-cadherin (1:1000, Abcam), N-cadherin (1:1000, Abcam), and Vimentin (1:1000, Abcam). β-Actin (1:2000, Abcam) was used as an internal control.

### Cell proliferation assay

Cell Counting Kit-8 (CCK-8, Dojondo, Tabaru, Japan) was used to measure cell proliferation. Cells were seeded into 96-well plates. The absorption values were measured at 24, 48, and 72 hours after shRNA transfection. The experiments were repeated three times, and the data are shown as the mean±standard deviation (SD).

### Colony formation assay

To determine long-term effects, the cells were seeded in a six-well plate. After 14 days, colonies were stained with crystal violet (Sigma-Aldrich, St. Louis, MO, USA), and the number of colonies was counted.

### Transwell migration and invasion assay

Migration and invasion were assessed using Transwell chambers. Cells in serum-free media were distributed into the inserts. Equal amounts of growth media were placed into the wells. After culturing overnight, the chamber membrane was stained with 50% methanol blue/ethanol overnight.

### Xenograft mouse model

Four-week-old BLAB/c nude mice were randomly divided into groups and received 5×10^5^ stable cell injections. Tumour volume was measured using a Vernier calliper. We injected approximately 5×10^4^ cells through the caudal vein to establish a lung metastasis model. After 2 weeks, live imaging was performed.

### Statistical analysis

All data analyses were performed with SPSS 20.0 statistical software. The χ^2^ test was used to analyse the relationships between categorical variables. The differences between groups were compared by Student’s *t*-test. Cox regression and Kaplan-Meier methods were used to analyse OS, and *p*<0.05 was considered to be a significant difference from the control.

## Supplementary Material

Supplementary Figure 1

## References

[1] Torre LA, Bray F, Siegel RL, Ferlay J, Lortet-Tieulent J, Jemal A. Global cancer statistics, 2012. CA Cancer J Clin. 2015; 65:87–108. 10.3322/caac.21262 25651787

[2] Ess SM, Herrmann C, Bouchardy C, Neyroud I, Rapiti E, Konzelmann I, Bordoni A, Ortelli L, Rohrmann S, Frick H, Mousavi M, Thürlimann B. Impact of subtypes and comorbidities on breast cancer relapse and survival in population-based studies. Breast. 2018; 41:151–58. 10.1016/j.breast.2018.07.011 30099326

[3] O’Brien KM, Mooney T, Fitzpatrick P, Sharp L. Screening status, tumour subtype, and breast cancer survival: a national population-based analysis. Breast Cancer Res Treat. 2018; 172:133–42. 10.1007/s10549-018-4877-9 30006795

[4] Sun Y, Bao W, Liu B, Caan BJ, Lane DS, Millen AE, Simon MS, Thomson CA, Tinker LF, Van Horn LV, Vitolins MZ, Snetselaar LG. Changes in Overall Diet Quality in Relation to Survival in Postmenopausal Women with Breast Cancer: Results from the Women’s Health Initiative. J Acad Nutr Diet. 2018; 118:1855–1863.e6. 10.1016/j.jand.2018.03.017 29859758

[5] Wang Z, Katsaros D, Biglia N, Shen Y, Fu Y, Loo LW, Jia W, Obata Y, Yu H. High expression of long non-coding RNA MALAT1 in breast cancer is associated with poor relapse-free survival. Breast Cancer Res Treat. 2018; 171:261–71. 10.1007/s10549-018-4839-2 29845475 PMC6488226

[6] Dayer R, Babashah S, Jamshidi S, Sadeghizadeh M. Upregulation of CXC chemokine receptor 4-CXC chemokine ligand 12 axis ininvasive breast carcinoma: A potent biomarker predicting lymph node metastasis. J Cancer Res Ther. 2018; 14:345–50. 10.1186/bcr627 29516917

[7] Mishra P, Chan DC. Mitochondrial dynamics and inheritance during cell division, development and disease. Nat Rev Mol Cell Biol. 2014; 15:634–46. 10.1038/nrm3877 25237825 PMC4250044

[8] Nasrallah CM, Horvath TL. Mitochondrial dynamics in the central regulation of metabolism. Nat Rev Endocrinol. 2014; 10:650–58. 10.1038/nrendo.2014.160 25200564

[9] Nunnari J, Suomalainen A. Mitochondria: in sickness and in health. Cell. 2012; 148:1145–59. 10.1016/j.cell.2012.02.035 22424226 PMC5381524

[10] Wang K, Zhang DL, Long B, An T, Zhang J, Zhou LY, Liu CY, Li PF. NFAT4-dependent miR-324-5p regulates mitochondrial morphology and cardiomyocyte cell death by targeting Mtfr1. Cell Death Dis. 2015; 6:e2007. 10.1038/cddis.2015.348 26633713 PMC4720883

[11] Musicco C, Cormio G, Pesce V, Loizzi V, Cicinelli E, Resta L, Ranieri G, Cormio A. Mitochondrial Dysfunctions in Type I Endometrial Carcinoma: Exploring Their Role in Oncogenesis and Tumor Progression. Int J Mol Sci. 2018; 19:19. 10.3390/ijms19072076 30018222 PMC6073675

[12] Chen H, Chan DC. Mitochondrial dynamics—fusion, fission, movement, and mitophagy—in neurodegenerative diseases. Hum Mol Genet. 2009; 18:R169–76. 10.1093/hmg/ddp326 19808793 PMC2758711

[13] Yu C, Wang Y, Peng J, Shen Q, Chen M, Tang W, Li X, Cai C, Wang B, Cai S, Meng X, Zou F. Mitochondrial calcium uniporter as a target of microRNA-340 and promoter of metastasis via enhancing the Warburg effect. Oncotarget. 2017; 8:83831–44. 10.18632/oncotarget.19747 29137386 PMC5663558

[14] Peiris-Pagès M, Bonuccelli G, Sotgia F, Lisanti MP. Mitochondrial fission as a driver of stemness in tumor cells: mDIVI1 inhibits mitochondrial function, cell migration and cancer stem cell (CSC) signalling. Oncotarget. 2018; 9:13254–75. 10.18632/oncotarget.24285 29568355 PMC5862576

[15] Kong B, Tsuyoshi H, Orisaka M, Shieh DB, Yoshida Y, Tsang BK. Mitochondrial dynamics regulating chemoresistance in gynecological cancers. Ann N Y Acad Sci. 2015; 1350:1–16. 10.1111/nyas.12883 26375862

[16] Fan S, Chen WX, Lv XB, Tang QL, Sun LJ, Liu BD, Zhong JL, Lin ZY, Wang YY, Li QX, Yu X, Zhang HQ, Li YL, et al. miR-483-5p determines mitochondrial fission and cisplatin sensitivity in tongue squamous cell carcinoma by targeting FIS1. Cancer Lett. 2015; 362:183–91. 10.1016/j.canlet.2015.03.045 25843291

[17] Wang J, Xie Y, Bai X, Wang N, Yu H, Deng Z, Lian M, Yu S, Liu H, Xie W, Wang M. Targeting dual specificity protein kinase TTK attenuates tumorigenesis of glioblastoma. Oncotarget. 2017; 9:3081–88. 10.18632/oncotarget.23152 29423030 PMC5790447

[18] Keysar SB, Eagles JR, Miller B, Jackson BC, Chowdhury FN, Reisinger J, Chimed TS, Le PN, Morton JJ, Somerset HL, Varella-Garcia M, Tan AC, Song JI, et al. Salivary Gland Cancer Patient-Derived Xenografts Enable Characterization of Cancer Stem Cells and New Gene Events Associated with Tumor Progression. Clin Cancer Res. 2018; 24:2935–43. 10.1158/1078-0432.CCR-17-3871 29555661 PMC6004240

[19] Monticone M, Panfoli I, Ravera S, Puglisi R, Jiang MM, Morello R, Candiani S, Tonachini L, Biticchi R, Fabiano A, Cancedda R, Boitani C, Castagnola P. The nuclear genes Mtfr1 and Dufd1 regulate mitochondrial dynamic and cellular respiration. J Cell Physiol. 2010; 225:767–76. 10.1002/jcp.22279 20568109

[20] Yeung KT, Yang J. Epithelial-mesenchymal transition in tumor metastasis. Mol Oncol. 2017; 11:28–39. 10.1002/1878-0261.12017 28085222 PMC5242415

[21] Zhao J, Zhang J, Yu M, Xie Y, Huang Y, Wolff DW, Abel PW, Tu Y. Mitochondrial dynamics regulates migration and invasion of breast cancer cells. Oncogene. 2013; 32:4814–24. 10.1038/onc.2012.494 23128392 PMC3911914

[22] Rehman J, Zhang HJ, Toth PT, Zhang Y, Marsboom G, Hong Z, Salgia R, Husain AN, Wietholt C, Archer SL. Inhibition of mitochondrial fission prevents cell cycle progression in lung cancer. FASEB J. 2012; 26:2175–86. 10.1096/fj.11-196543 22321727 PMC3336787

[23] Sonbul SN, Aleskandarany MA, Kurozumi S, Joseph C, Toss MS, Diez-Rodriguez M, Nolan CC, Mukherjee A, Martin S, Caldas C, Ellis IO, Green AR, Rakha EA. Saccharomyces cerevisiae-like 1 (SEC14L1) is a prognostic factor in breast cancer associated with lymphovascular invasion. Mod Pathol. 2018; 31:1675–82. 10.1038/s41379-018-0092-9 29955149

[24] Claessens AK, Bos ME, Lopez-Yurda M, Bouma JM, Rademaker-Lakhai JM, Honkoop AH, de Graaf H, van Druten E, van Warmerdam LJ, van der Sangen MJ, Tjan-Heijnen VC, Erdkamp FL, and Dutch Breast Cancer Research Group (BOOG). Intermittent versus continuous first-line treatment for HER2-negative metastatic breast cancer: the Stop & Go study of the Dutch Breast Cancer Research Group (BOOG). Breast Cancer Res Treat. 2018; 172:413–23. 10.1007/s10549-018-4906-8 30121808

[25] Zhou H, Zhu P, Wang J, Zhu H, Ren J, Chen Y. Pathogenesis of cardiac ischemia reperfusion injury is associated with CK2α-disturbed mitochondrial homeostasis via suppression of FUNDC1-related mitophagy. Cell Death Differ. 2018; 25:1080–93. 10.1038/s41418-018-0086-7 29540794 PMC5988750

[26] Lin S, Hoffmann K, Gao C, Petrulionis M, Herr I, Schemmer P. Melatonin promotes sorafenib-induced apoptosis through synergistic activation of JNK/c-jun pathway in human hepatocellular carcinoma. J Pineal Res. 2017; 62:62. 10.1111/jpi.12398 28178378

[27] Szabadkai G, Duchen MR. Mitochondria: the hub of cellular Ca2+ signaling. Physiology (Bethesda). 2008; 23:84–94. 10.1152/physiol.00046.2007 18400691

[28] Lima AR, Santos L, Correia M, Soares P, Sobrinho-Simões M, Melo M, Máximo V. Dynamin-Related Protein 1 at the Crossroads of Cancer. Genes (Basel). 2018; 9:9. 10.3390/genes9020115 29466320 PMC5852611

[29] Sheridan C, Martin SJ. Mitochondrial fission/fusion dynamics and apoptosis. Mitochondrion. 2010; 10:640–48. 10.1016/j.mito.2010.08.005 20727425

[30] Youle RJ, van der Bliek AM. Mitochondrial fission, fusion, and stress. Science. 2012; 337:1062–65. 10.1126/science.1219855 22936770 PMC4762028

[31] Ashrafi G, Schwarz TL. The pathways of mitophagy for quality control and clearance of mitochondria. Cell Death Differ. 2013; 20:31–42. 10.1038/cdd.2012.81 22743996 PMC3524633

[32] Vander Heiden MG, DeBerardinis RJ. Understanding the Intersections between Metabolism and Cancer Biology. Cell. 2017; 168:657–69. 10.1016/j.cell.2016.12.039 28187287 PMC5329766

[33] Cassim S, Raymond VA, Dehbidi-Assadzadeh L, Lapierre P, Bilodeau M. Metabolic reprogramming enables hepatocarcinoma cells to efficiently adapt and survive to a nutrient-restricted microenvironment. Cell Cycle. 2018; 17:903–16. 10.1080/15384101.2018.1460023 29633904 PMC6056217

[34] Cassim S, Raymond VA, Lacoste B, Lapierre P, Bilodeau M. Metabolite profiling identifies a signature of tumorigenicity in hepatocellular carcinoma. Oncotarget. 2018; 9:26868–83. 10.18632/oncotarget.25525 29928490 PMC6003570

[35] Lacoste B, Raymond VA, Cassim S, Lapierre P, Bilodeau M. Highly tumorigenic hepatocellular carcinoma cell line with cancer stem cell-like properties. PLoS One. 2017; 12:e0171215. 10.1371/journal.pone.0171215 28152020 PMC5289561

[36] Dongre A, Weinberg RA. New insights into the mechanisms of epithelial-mesenchymal transition and implications for cancer. Nat Rev Mol Cell Biol. 2019; 20:69–84. 10.1038/s41580-018-0080-4 30459476

[37] Morigi C. Highlights from the 15th St Gallen International Breast Cancer Conference 15-18 March, 2017, Vienna: tailored treatments for patients with early breast cancer. Ecancermedicalscience. 2017; 11:732. 10.3332/ecancer.2017.732 28491135 PMC5406222

