# Correction for: Mitochondrial fission regulator 2 (MTFR2) promotes growth, migration, invasion and tumour progression in breast cancer cells

**DOI:** 10.18632/aging.102758

**Published:** 2020-01-08

**Authors:** Guanming Lu, Lai Yuanhui, Tiantian Wang, Weihao Lin, Jinlan Lu, Yanfei Ma, Yongcheng Chen, Haiqing Ma, Ruilei Liu, Jie Li

**Affiliations:** 1 Department of Breast and Thyroid Surgery, Affiliated Hospital of Youjiang Medical University for Nationalities, Basie, Guangxi, China; 2 Department of Breast and Thyroid Surgery, Eastern Hospital of the First Affiliated Hospital of Sun Yat-sen University, Guangzhou, Guangdong, China; 3 Department of Breast and Thyroid Surgery, Shandong Provincial Hospital Affiliated to Shandong University, Ji’nan, Shandong, China; 4 Department of Breast and Thyroid Surgery, First Affiliated Hospital of Sun Yat-sen University, Guangzhou, Guangdong, China; 5 Department of Stomatology, Affiliated Hospital of Youjiang Medical University for Nationalities, Basie, Guangxi, China; 6 Department of Oncology, Fifth Affiliated Hospital of Sun Yat-sen University, Zhuhai, Guangdong, China; 7 Department of Breast and Thyroid Surgery, Third Affiliated Hospital of Sun Yat-sen University, Guangzhou, Guangdong, China; 8 Division of Thyroid and Parathyroid Endocrine Surgery, Massachusetts Eye and Ear Infirmary, Harvard Medical School, Boston, MA 02114, USA

**Keywords:** correction

**This article has been corrected:** The authors requested to replace Figure 3 and Figure 6. The mistakes of these figures are described below:

**Figure 3:** the Westernblot of SDHB in Figure3B of MCF-7 flipped horizontally.

**Figure 6:** the Westernblot of Cytc in Figure6B of MDA-231 was identical to Uqcrfs1 due to the layout mistakes.

These corrections do not change any of the conclusions of the publication. The corrected Figure 3 and Figure 6 are provided below.

**Figure 3:**
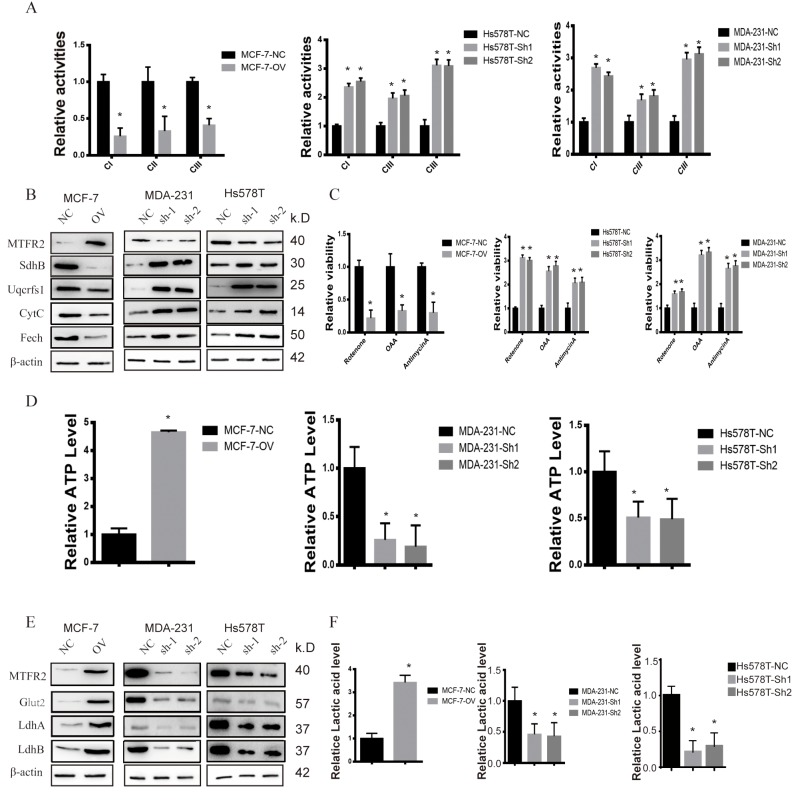
**MTFR promotes the glycolysis of BC. **(**A**) The relative activities of the CI CII and CIII of different cell lines (Student’s two one-tailed paired test * p<0.05). (**B**) Western blot of OXPHOS markers of different cell lines. (**C**) The relative viability of different cell lines treated with different inhibitors (Student’s two one-tailed paired test * p<0.05). (**D**) The relative ATP level of different cell lines (Student’s two one-tailed paired test * p<0.05). (**E**) Western blot of glycolysis markers of different cell lines. (**F**) The relative lactic acid level of different cell lines (Student’s two one-tailed paired test * p<0.05).

**Figure 6:**
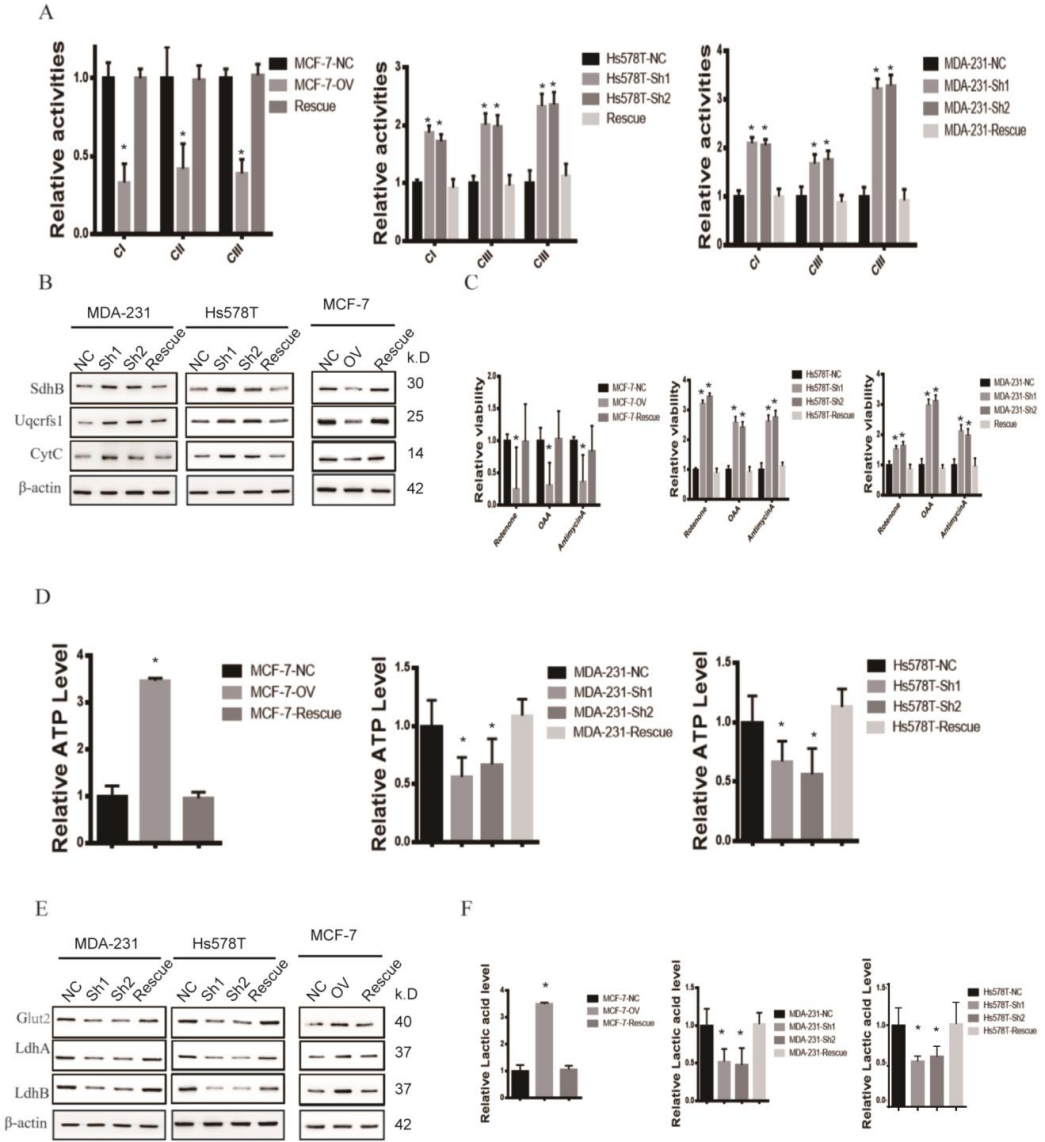
**MTFR promotes the glycolysis of BC in a HIF1α- and HIF2α-dependent manner. **(**A**) The relative activities of the CI CII and CIII of different cell lines (Student’s two one-tailed paired test * p<0.05). (**B**) Western blot of OXPHOS markers of different cell lines. (**C**) The relative viability of different cell lines treated with different inhibitors (Student’s two one-tailed paired test * p<0.05). (**D**) The relative ATP level of different cell lines (Student’s two one-tailed paired test * p<0.05). (**E**) Western blot of glycolysis markers of different cell lines. (**F**) The relative lactic acid level of different cell line (Student’s two one-tailed paired test * p<0.05).

Original article: Aging. 2019; 11:10203–10219. https://doi.org/10.18632/aging.102442.

