# Supplementary material for: Mitochondrial fission regulator 2 (MTFR2) promotes growth, migration, invasion and tumour progression in breast cancer cells
Source: Aging (Albany NY). 2019 Nov 18;11(22):10203–19. doi: 10.18632/aging.102442 (PMC6914410; doi:10.18632/aging.102442)
Supplement: Supplementary Figure 1 [file aging-11-102442-s001..pdf]

SUPPLEMENTARY FIGURE

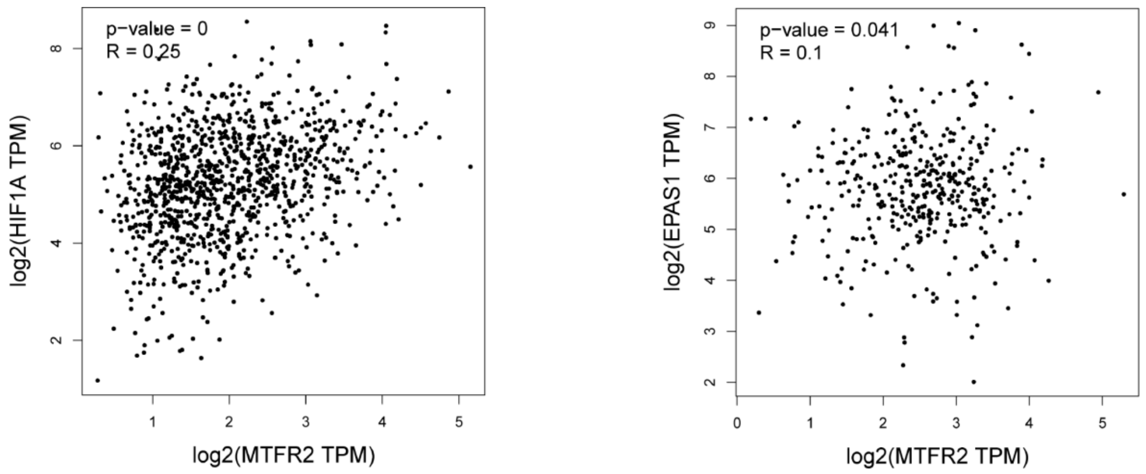

Supplementary Figure 1. The correlation between MTFR2 and HIF1 α, HIF2 α.
